# Supplementary material for: An obesogenic FTO allele causes accelerated development, growth and insulin resistance in human skeletal muscle cells
Source: Nat Commun. 2025 Mar 7;16:1645. doi: 10.1038/s41467-024-53820-2 (PMC11889117; doi:10.1038/s41467-024-53820-2)
Supplement: Supplementary file 1 — Supplementary Information [file 41467_2024_53820_MOESM1_ESM.pdf]

Supplementary Fig. 1

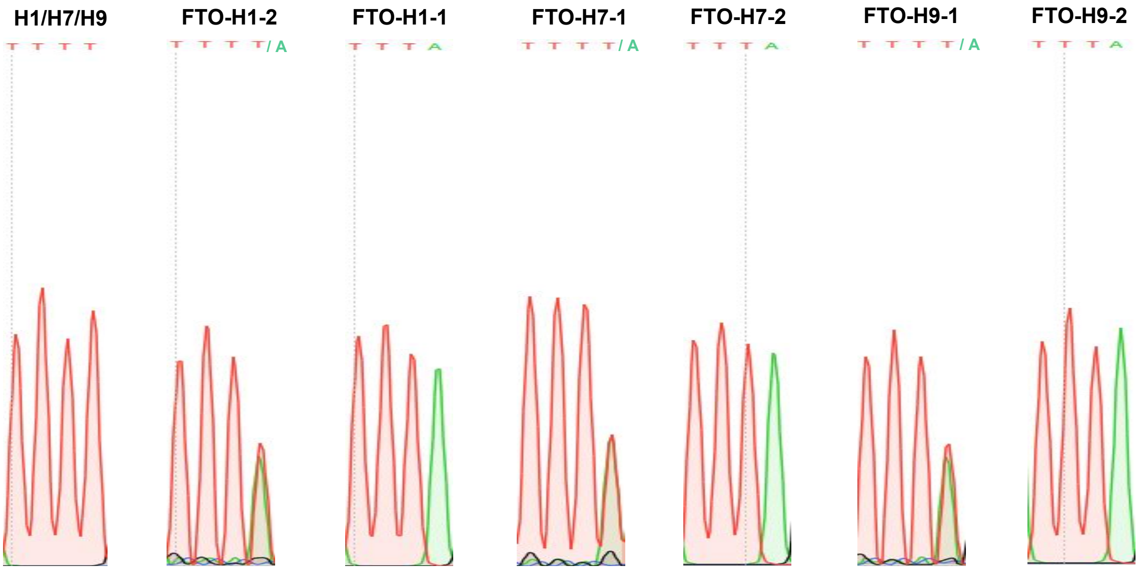

Supplementary Fig. 1.

PCR-based Sanger sequencing of FTO$^{rs9939609\text{-}TT}$ hESC lines: H1, H7, H9 and FTO$^{rs9939609\text{-}A}$ hESC lines. FTO-H1-2, FTO-H7-1, FTO-H9-1 were heterozygous, and FTO-H1-1, FTO-H7-2, FTO-H9-2 were homozygous for rs9939609-A.

Supplementary Fig. 2

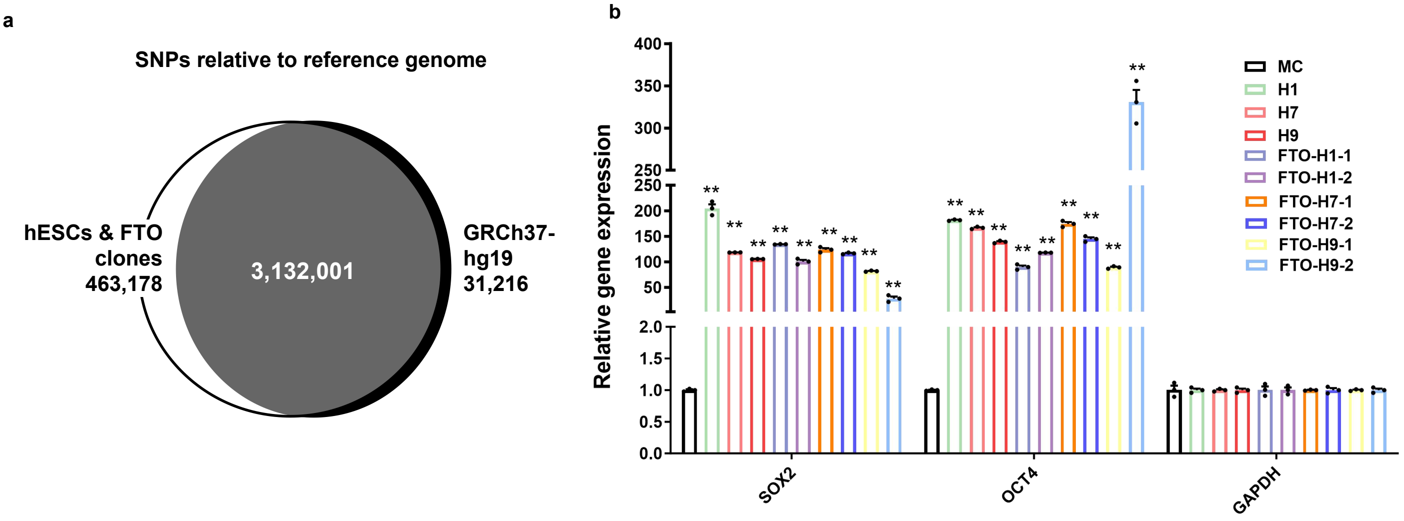

Supplementary Fig. 2.

a, Genomic stability of FTO$^{rs9939609\text{-}TT}$ hESC lines and FTO$^{rs9939609\text{-}A}$ hESC clones, according to deep sequencing for SNPs, in comparison with the GRCh37-hg19 human reference genome. **b,** Normal pluripotency of hESCs lines and FTO$^{rs9939609\text{-}A}$ hESC clones by qPCR, n = 3 independent experiments. Data are presented as mean + SEM. P values were calculated by two-tailed unpaired t-test. $^{*}P < 0.05$, $^{**}P < 0.01$. Source data are provided as a Source Data file.

Supplementary Fig. 3

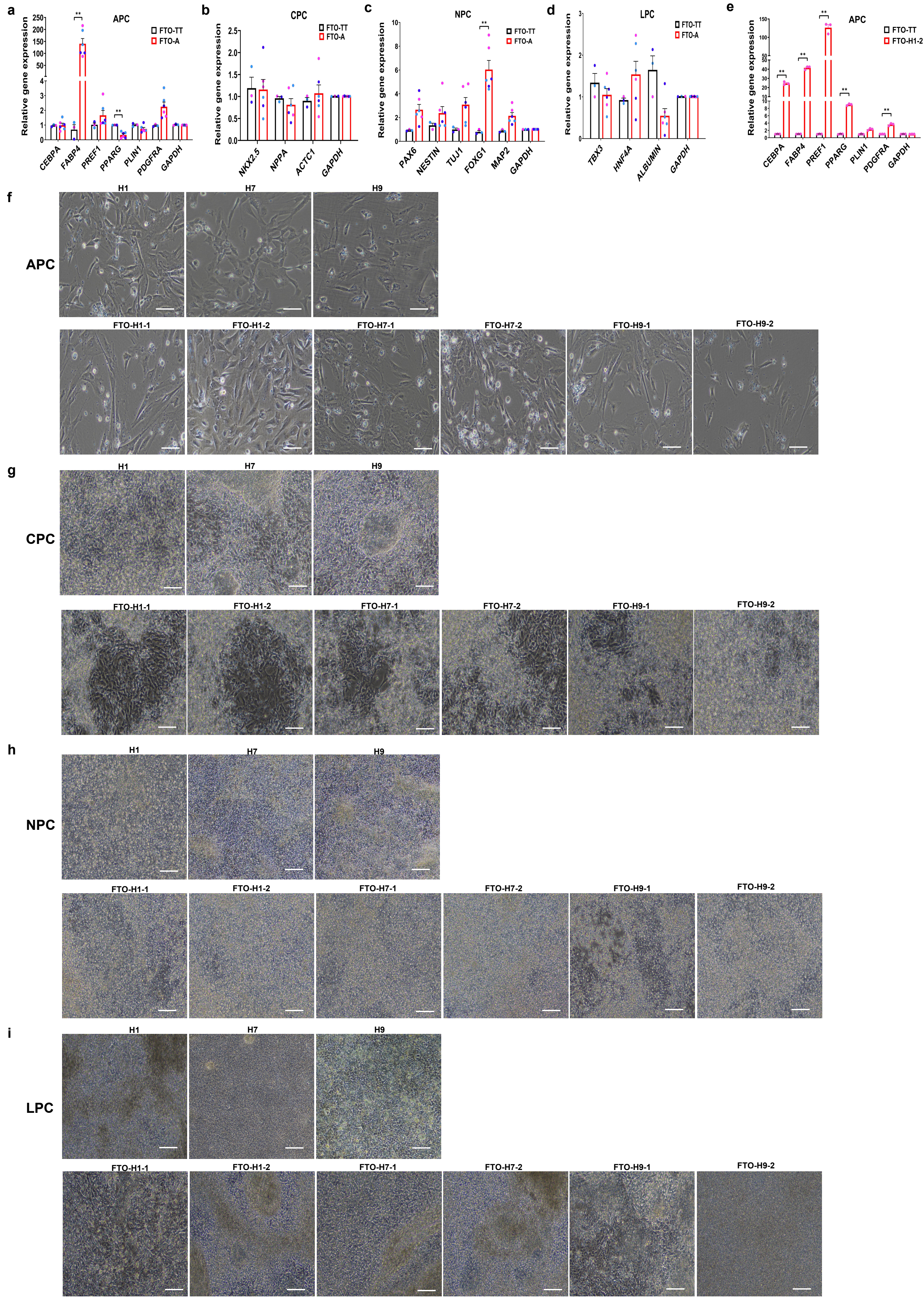

Supplementary Fig. 3.

a, Quantitative RT-PCR of CCAAT Enhancer Binding Protein Alpha (*CEBPA*), Fatty Acid Binding Protein 4 (*FABP4*), Preadipocyte factor 1 (*PREF1*), Peroxisome Proliferator Activated Receptor Gamma (*PPARG*), Perilipin 1 (*PLIN1*) and Platelet derived growth factor receptor alpha (*PDGFRA*) in $FTO^{rs9939609\text{-}TT}$ and $FTO^{rs9939609\text{-}A}$ cells after Adipose tissue differentiation, *n* = 3 biologically independent samples. **b,** Quantitative RT-PCR of NK2 Homeobox 5 (*NKX2.5*), Natriuretic peptide A (*NPPA*), Actin alpha cardiac muscle 1 (*ACTC1*) in $FTO^{rs9939609\text{-}TT}$ and $FTO^{rs9939609\text{-}A}$ cells after Cardiac tissue differentiation, *n* = 3 biologically independent samples. **c,** Quantitative RT-PCR of Paired Box 6 (*PAX6*), *NESTIN*, Neuronal Class III β-Tubulin (*TUJ1*), Forkhead Box G1 (*FOXG1*) and Microtubule Associated Protein 2 (*MAP2*) in $FTO^{rs9939609\text{-}TT}$ and $FTO^{rs9939609\text{-}A}$ cells after Neural tissue differentiation, *n* = 3 biologically independent samples. **d,** Quantitative RT-PCR of T-Box Transcription Factor 3 (*TBX3*), Hepatocyte Nuclear Factor 4 Alpha (*HNF4A*) and *ALBUMIN* in $FTO^{rs9939609\text{-}TT}$ and $FTO^{rs9939609\text{-}A}$ cells after Liver tissue differentiation, *n* = 3 biologically independent samples. **e,** Quantitative RT-PCR of CCAAT Enhancer Binding Protein Alpha (*CEBPA*), Fatty Acid Binding Protein 4 (*FABP4*), Preadipocyte factor 1 (*PREF1*), Peroxisome Proliferator Activated Receptor Gamma (*PPARG*), Perilipin 1 (*PLIN1*) and Platelet derived growth factor receptor alpha (*PDGFRA*) in $FTO^{rs9939609\text{-}TT}$ (H1) and $FTO^{rs9939609\text{-}A}$ cell (FTO-H1-2) after Adipose tissue differentiation, *n* = 3 independent experiments. **f-i,** Images of adipose, cardiac, neural and liver progenitors, *n* = 3 independent experiments. Scale bars, 100 μm. H1 hESC line coloured in purple, H7 hESC line coloured in light blue and H9 hESC line coloured in dark blue. Data are presented as mean + SEM. P values were calculated by two-tailed unpaired t-test. *P < 0.05, **P < 0.01. Source data are provided as a Source Data file.

Supplementary Fig. 4

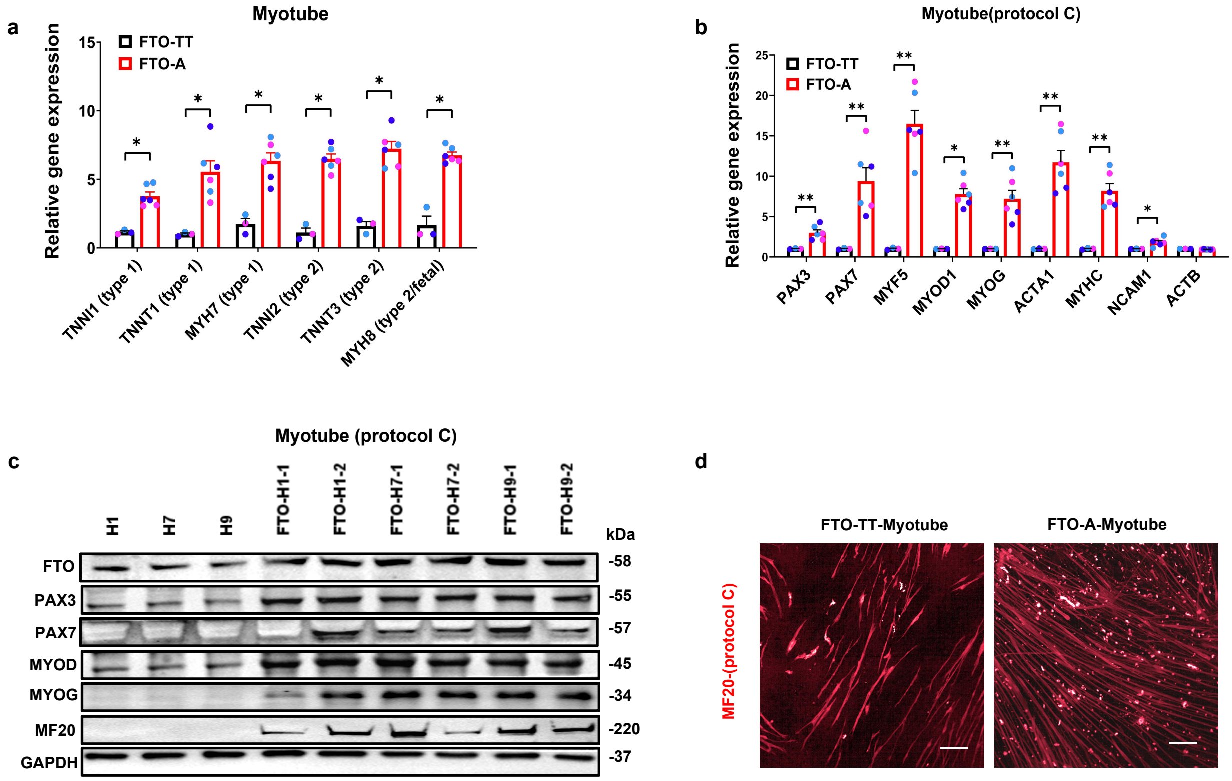

Supplementary Fig. 4.

a, Quantitative RT-PCR of troponin I1, slow skeletal type (*TNNI1*), troponin T1, slow skeletal type (*TNNT1*), myosin heavy chain 7 (*MYH7*), troponin I2, fast skeletal type (*TNNI2*), troponin T3, fast skeletal type (*TNNT3*), myosin heavy chain 8 (*MYH8*) in myotubes derived using the efficient protocol according to Loh et al., 2016[105]. *n* = 3 biologically independent samples. **b,** Quantitative RT-PCR of myogenic markers in FTO$^{rs9939609\text{-}TT}$-myotubes and FTO$^{rs9939609\text{-}A}$-myotubes derived using another protocol108. Paired box 3 (*PAX3*), Paired box 7 (*PAX7*), Myogenic factor 5 (*MYF5*), Myogenic differentiation 1 (*MYOD1*), Myogenin (*MYOG*), Skeletal muscle actin alpha 1 (*ACTA1*), Myosin heavy chain (*MYHC*), Neural cell adhesion molecule 1 (*NCAM1*), Actin Beta (*ACTB*), *n* = 3 biologically independent samples. **c,** Western blot quantification of FTO, PAX3, PAX7, MYOD1, MYOG, MF20 and GAPDH protein abundance in FTO$^{rs9939609\text{-}TT}$-myotubes and FTO$^{rs9939609\text{-}A}$-myotubes using another protocol[108]. **d,** Representative images of the FTO$^{rs9939609\text{-}TT}$-myotubes and FTO$^{rs9939609\text{-}A}$-myotubes derived using another protocol108. Scale bars, 100 μm, *n* = 3 biologically independent samples. H1 hESC line coloured in purple, H7 hESC line coloured in light blue and H9 hESC line coloured in dark blue. Data are presented as mean + SEM. P values were calculated by two-tailed unpaired t-test. *P < 0.05, **P < 0.01. Source data are provided as a Source Data file.

Supplementary Fig. 5

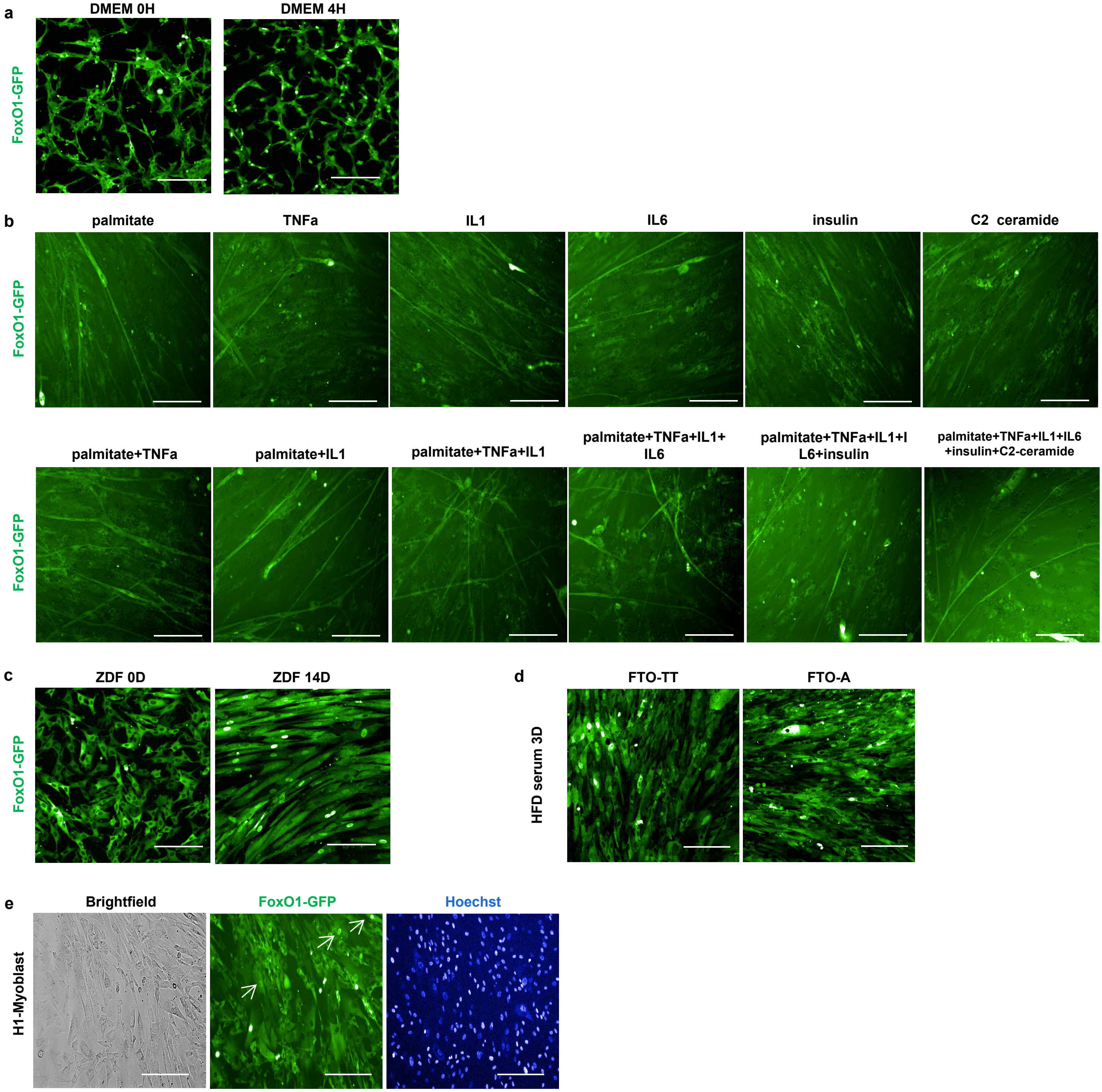

Supplementary Fig. 5.

a, Representative images of FoxO1-GFP myocytes under conditions of serum starvation after 4 hours. Scale bars, 200 µm, *n* = 3 biologically independent samples. **b,** Representative images of FoxO1-GFP myotubes exposed to 1% B27 and 0.3mM palmitate, 30ng/ml TNFa, 20ng/ml IL1, 100ng/ml IL6, 2µM insulin, 20µM C2 ceramide and combinations there of 14 days. Scale bars, 200 µm, *n* = 3 biologically independent samples. **c,** Representative images of FoxO1-GFP myocytes exposed to 1% ZDF rat serum at day 0 and day 14. Scale bars, 200 µm, *n* = 3 biologically independent samples. **d,** Representative images of FTO$^{rs9939609\text{-}TT}$-myocytes and FTO$^{rs9939609\text{-}A}$-myocytes exposed to 1% HFD mouse serum at day3. Scale bars, 200 µm, *n* = 3 biologically independent samples. **e,** Representative images of senescent hESC-myoblasts FoxO1-GFP localization. Scale bars, 200 µm, *n* = 3 biologically independent samples. Data are presented as mean + SEM. P values were calculated by two-tailed unpaired t-test. *P < 0.05, **P < 0.01. Source data are provided as a Source Data file.

Supplementary Fig. 6

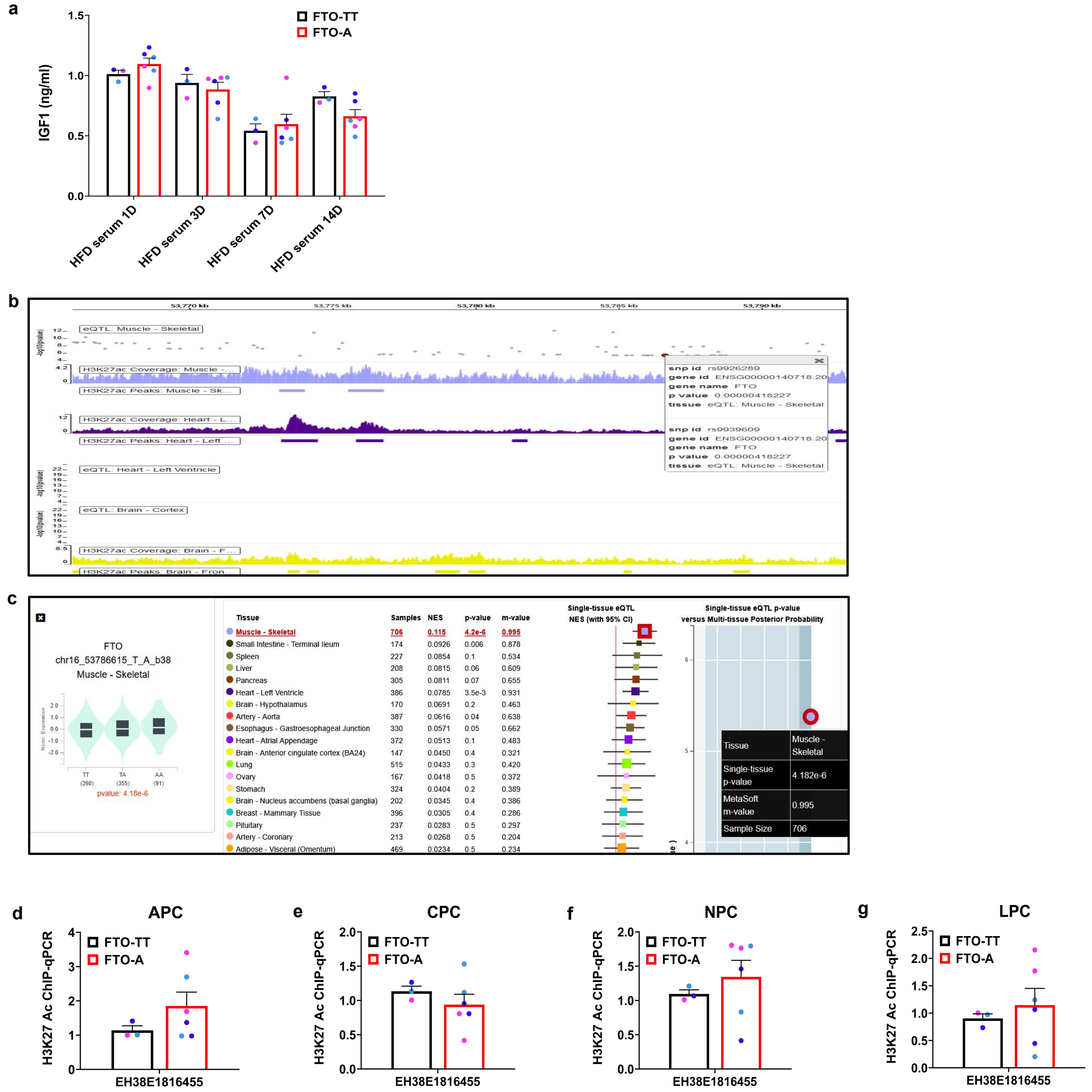

Supplementary Fig. 6.

a, ELISA analysis for IGF1 of FTO$^{rs9939609\text{-}TT}$-myotubes and FTO$^{rs9939609\text{-}A}$-myotubes after exposure to 1% HFD mouse serum for 1 day, 3 days, 7 days, and 14 days, *n* = 3 biologically independent samples. **b-c,** Multi-tissue eQTL comparison of the genomic region near rs9939609. **d-g,** H3K27ac ChIP-qPCR of the EH38E1816455 enhancer near rs9939609 in FTO$^{rs9939609\text{-}TT}$ and FTO$^{rs9939609\text{-}A}$ adipose, cardiac, neural and liver progenitors, *n* = 3 biologically independent samples. H1 hESC line coloured in purple, H7 hESC line coloured in light blue and H9 hESC line coloured in dark blue. Data are presented as mean + SEM. P values were calculated by two-tailed unpaired t-test. *P < 0.05, **P < 0.01. Source data are provided as a Source Data file.

Supplementary Fig. 7

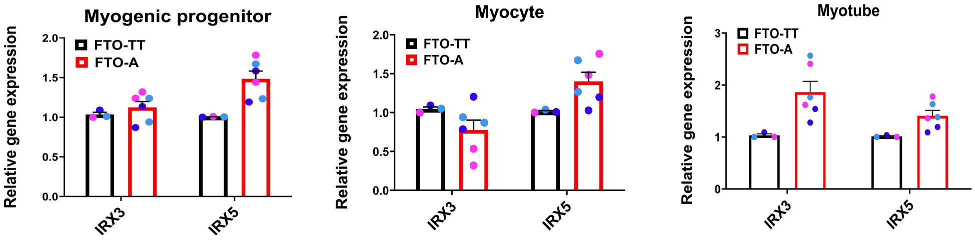

Supplementary Fig.7.

Quantitative RT-PCR for Iroquois Homeobox 3 (*IRX3*) and Iroquois Homeobox 5 (*IRX5*) in FTO$^{rs9939609\text{-}TT}$ and FTO$^{rs9939609\text{-}A}$ myogenic progenitors, myocytes and myotubes, *n* = 3 biologically independent samples. H1 hESC line coloured in purple, H7 hESC line coloured in light blue and H9 hESC line coloured in dark blue. Data are presented as mean + SEM. P values were calculated by two-tailed unpaired t-test. *P < 0.05, **P < 0.01. Source data are provided as a Source Data file.

Supplementary Fig. 8

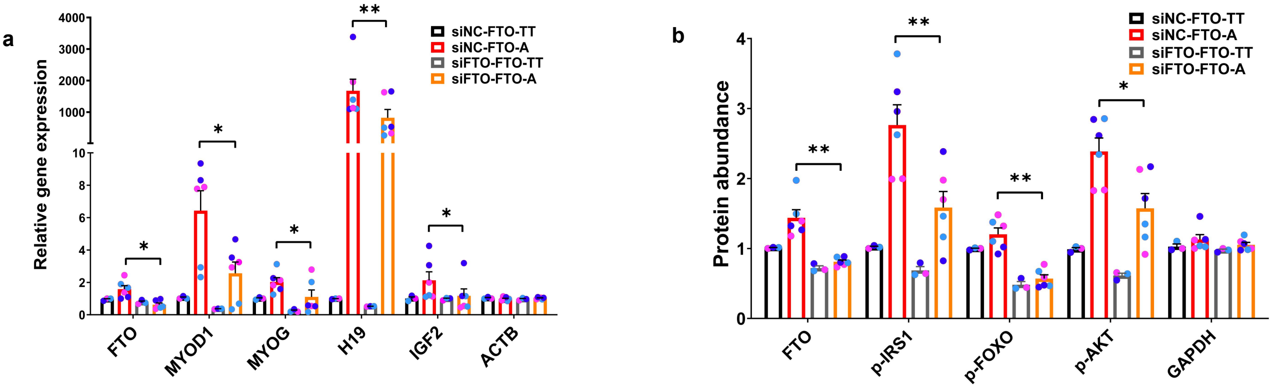

Supplementary Fig. 8.

a, Quantitative RT-PCR for *FTO, MYOD1, MYOG, H19, IGF2* and *ACTB*. siNC: knockdown negative control; siFTO: knockdown of *FTO*, *n* = 3 biologically independent samples. **b,** Quantification of the abundance of myogenic and insulin signaling proteins,*n* = 3 biologically independent samples. siNC: knockdown negative control; siFTO: knockdown of *FTO*. H1 hESC line coloured in purple, H7 hESC line coloured in light blue and H9 hESC line coloured in dark blue. Data are presented as mean + SEM. P values were calculated by two-tailed unpaired t-test. *P < 0.05, **P < 0.01. Source data are provided as a Source Data file.
