# Supplementary material for: An obesogenic FTO allele causes accelerated development, growth and insulin resistance in human skeletal muscle cells
Source: Nat Commun. 2025 Mar 7;16:1645. doi: 10.1038/s41467-024-53820-2 (PMC11889117; doi:10.1038/s41467-024-53820-2)
Supplement: Supplementary file 3 — Description of Additional Supplementary Files [file 41467_2024_53820_MOESM3_ESM.pdf]

# Description of Additional Supplementary Files

## Supplementary Movie Legends:

**Supplementary Movie 1.** 2D FTOrs9939609 - TT myotubes. Time, 20s.

**Supplementary Movie 2.** 2D FTOrs9939609 - A myotubes. Time, 20s.

**Supplementary Movie 3.** 3D FTOrs9939609 - TT organoid. Time, 30s.

**Supplementary Movie 4.** 3D FTOrs9939609- A organoid. Time, 30s.

## Supplementary Data Legends:

**Supplementary Data 1:** Primers, RNAi and Crispr used in this study

**Supplementary Data 2:** Reagent and Resource used in this study
