# Supplementary material for: An obesogenic FTO allele causes accelerated development, growth and insulin resistance in human skeletal muscle cells
Source: Nat Commun. 2025 Mar 7;16:1645. doi: 10.1038/s41467-024-53820-2 (PMC11889117; doi:10.1038/s41467-024-53820-2)
Supplement: Supplementary file 10 — Reporting Summary [file 41467_2024_53820_MOESM10_ESM.pdf]

nature portfolio

Corresponding author(s): Ng Shyh-Chang

Last updated by author(s): Oct 14, 2024

Reporting Summary

Nature Portfolio wishes to improve the reproducibility of the work that we publish. This form provides structure for consistency and transparency in reporting. For further information on Nature Portfolio policies, see our Editorial Policies and the Editorial Policy Checklist.

Statistics

For all statistical analyses, confirm that the following items are present in the figure legend, table legend, main text, or Methods section.

n/a	Confirmed	
☐	☒	The exact sample size (*n*) for each experimental group/condition, given as a discrete number and unit of measurement
☐	☒	A statement on whether measurements were taken from distinct samples or whether the same sample was measured repeatedly
☐	☒	The statistical test(s) used AND whether they are one- or two-sided<br>*Only common tests should be described solely by name; describe more complex techniques in the Methods section.*
☒	☐	A description of all covariates tested
☐	☒	A description of any assumptions or corrections, such as tests of normality and adjustment for multiple comparisons
☐	☒	A full description of the statistical parameters including central tendency (e.g. means) or other basic estimates (e.g. regression coefficient) AND variation (e.g. standard deviation) or associated estimates of uncertainty (e.g. confidence intervals)
☐	☒	For null hypothesis testing, the test statistic (e.g. *F*, *t*, *r*) with confidence intervals, effect sizes, degrees of freedom and *P* value noted<br>*Give P values as exact values whenever suitable.*
☒	☐	For Bayesian analysis, information on the choice of priors and Markov chain Monte Carlo settings
☒	☐	For hierarchical and complex designs, identification of the appropriate level for tests and full reporting of outcomes
☐	☒	Estimates of effect sizes (e.g. Cohen's *d*, Pearson's *r*), indicating how they were calculated

Our web collection on statistics for biologists contains articles on many of the points above.

Software and code

Policy information about availability of computer code

Data collection	PerkinElmer Operetta CLS.
Data analysis	GraphPad Prism version 10.0.2; Image Jversion 1.8.0.

For manuscripts utilizing custom algorithms or software that are central to the research but not yet described in published literature, software must be made available to editors and reviewers. We strongly encourage code deposition in a community repository (e.g. GitHub). See the Nature Portfolio guidelines for submitting code & software for further information.

Data

Policy information about availability of data

All manuscripts must include a data availability statement. This statement should provide the following information, where applicable:

- Accession codes, unique identifiers, or web links for publicly available datasets
- A description of any restrictions on data availability
- For clinical datasets or third party data, please ensure that the statement adheres to our policy

All data are available in the main text or the supplementary materials. Source data are provided with this paper. All the sequencing data generated in this study have been deposited in the NCBI's Gene Expression Omnibus data bank under accession code GSE262261. Any additional information is available upon request to the corresponding author (Ng Shyh-Chang, huangsq@ioz.ac.cn).

Research involving human participants, their data, or biological material

Policy information about studies with human participants or human data. See also policy information about sex, gender (identity/presentation), and sexual orientation and race, ethnicity and racism.

Reporting on sex and gender	This study did not involve human participants.
Reporting on race, ethnicity, or other socially relevant groupings	This study did not involve human participants.
Population characteristics	This study did not involve human participants.
Recruitment	This study did not involve human participants.
Ethics oversight	This study did not involve human participants.

Note that full information on the approval of the study protocol must also be provided in the manuscript.

Field-specific reporting

Please select the one below that is the best fit for your research. If you are not sure, read the appropriate sections before making your selection.

☒ Life sciences ☐ Behavioural & social sciences ☐ Ecological, evolutionary & environmental sciences

For a reference copy of the document with all sections, see nature.com/documents/nr-reporting-summary-flat.pdf

Life sciences study design

All studies must disclose on these points even when the disclosure is negative.

Sample size	No statistical test was used to predetermine sample size. Sample size was based on experimental feasibility, sample availability, and necessary to obtain definitive results.
Data exclusions	No data were excluded.
Replication	Experiments were repeated with the same conditions and obtained similar results. The numbers of repeats were indicated in figure legends.
Randomization	All samples were randomly allocated.
Blinding	Blinds are placed on all participants in the experiment, including researchers, technicians, data analysts, and evaluators.

Reporting for specific materials, systems and methods

We require information from authors about some types of materials, experimental systems and methods used in many studies. Here, indicate whether each material, system or method listed is relevant to your study. If you are not sure if a list item applies to your research, read the appropriate section before selecting a response.

Materials & experimental systems

n/a	Involved in the study	
☐	☒	Antibodies
☐	☒	Eukaryotic cell lines
☒	☐	Palaeontology and archaeology
☐	☒	Animals and other organisms
☒	☐	Clinical data
☒	☐	Dual use research of concern
☒	☐	Plants

Methods

n/a	Involved in the study	
☒	☐	ChIP-seq
☒	☐	Flow cytometry
☒	☐	MRI-based neuroimaging

Antibodies

Antibodies used	anti-FTO (Abcam, ab94482, 1:1000 for WB);<br>anti-PAX3 (DSHB, AB528426, 1:120 for WB);<br>anti-PAX7 (DSHB, AB528428,1:60 for WB);

	anti-MYOD1 (Santa Cruz, sc-377460, 1:200 for WB);<br>anti-MYOG (Santa Cruz, sc-52903, 1:200 for WB);<br>anti-MF20 (DSHB, AB2147781, 1:600 for WB);<br>anti-pIRS1 (CST, 2385S, 1:1000 for WB);<br>anti-pFOXO1 (CST, 9461S, 1:1000 for WB);<br>anti-pAKT (CST 4060S, 1:1000 for WB);<br>anti-GAPDH (CST, 2118S, 1:1000 for WB);<br>anti-mouse IgG HRP (CST, 7076S, 1:1000 for WB);<br>anti-rabbit IgG HRP (CST, 7074S, 1:1000 for WB);<br>anti-m6A (Abcam, ab151230, 2 μg/ml for MeRIP-RT-qPCR);<br>anti-IGF2 (R&D Systems, AF-292-NA, 0.8 μg/ml for Inhibition of IGF2);<br>anti-Acetyl-Histone H3 (Lys27) (CST, 8173S, 1:100 for ChIP-RT-qPCR).
Validation	anti-FTO (Abcam, ab94482, https://www.abcam.cn/products/primary-antibodies/fto-antibody-ab94482.html);<br>anti-PAX3 (DSHB, AB528426, https://dshb.biology.uiowa.edu/Pax3);<br>anti-PAX7 (DSHB, AB528428,https://dshb.biology.uiowa.edu/PAX7);<br>anti-MYOD1 (Santa Cruz, sc-377460, https://www.scbt.com/zh/p/myod-antibody-g-1);<br>anti-MYOG (Santa Cruz, sc-52903, https://www.scbt.com/zh/p/myogenin-antibody-5fd);<br>anti-MF20 (DSHB, AB2147781, https://dshb.biology.uiowa.edu/MF-20);<br>anti-pIRS1 (CST, 2385S, https://www.cellsignal.cn/products/primary-antibodies/phospho-irs-1-ser1101-antibody/2385);<br>anti-pFOXO1 (CST, 9461S, https://www.cellsignal.cn/products/primary-antibodies/phospho-foxo1-ser256-antibody/9461);<br>anti-pAKT (CST 4060S, https://www.cellsignal.cn/products/primary-antibodies/phospho-akt-ser473-d9e-xp-174-rabbit-mab/4060);<br>anti-GAPDH (CST, 2118S, https://www.cellsignal.cn/products/primary-antibodies/gapdh-14c10-rabbit-mab/2118);<br>anti-mouse IgG HRP (CST, 7076S, https://www.cellsignal.cn/products/secondary-antibodies/anti-mouse-igg-hrp-linked-antibody/7076);<br>anti-rabbit IgG HRP (CST, 7074S, https://www.cellsignal.cn/products/secondary-antibodies/anti-rabbit-igg-hrp-linked-antibody/7074);<br>anti-m6A (Abcam, ab151230, https://www.abcam.cn/products/primary-antibodies/n6-methyladenosine-m6a-antibody-ab151230.html);<br>anti-IGF2 (R&D Systems, AF-292-NA, https://www.rndsystems.com/cn/products/human-igf-ii-igf2-antibody_af-292-na);<br>anti-Acetyl-Histone H3 (Lys27) (CST, 8173S, https://www.cellsignal.cn/products/primary-antibodies/acetyl-histone-h3-lys27-d5e4-xp-174-rabbit-mab/8173).

Eukaryotic cell lines

Policy information about cell lines and Sex and Gender in Research

Cell line source(s)	H1, H7, H9 human ESCs were purchased from WiCell in Wisconsin (WA01; WA07; WA09; WiCell, USA).
Authentication	Fresh or frozen cleavage stage human embryos, produced by in vitro fertilization (IVF) for clinical purposes, were donated by individuals after informed consent and after institutional review board approval. Embryos were cultured to the blastocyst stage, and ES cell lines originating from separate embryos were derived. H1 had a normal XY karyotype, and H7 and H9 had a normal XX karyotype.The cell line was obtained from suppliers. Cell authentication is based on their morphology, growth conditions and specific gene expression.
Mycoplasma contamination	We routinely check for mycoplasma in the lab and the cells were confirmed mycoplasma free at time of analysis.
Commonly misidentified lines<br>(See ICLAC register)	No commonly misidentified cell lines were used in this study.

Animals and other research organisms

Policy information about studies involving animals; ARRIVE guidelines recommended for reporting animal research, and Sex and Gender in Research

Laboratory animals	High fat diet (HFD) fed mouse (male, 19-week-old) were purchased from Vital River (Beijing, China). To generate DIO models, HF diet (60% kcal/fat, D12492, Research Diets) were introduced at 6- week-old and fed for 13 consecutive weeks before purchase. All mice were housed in a pathogen-free room conditioned at 20 – 22 ? with alternating 12 h cycles of light and dark, and with free access to pellet food and water.
Wild animals	This study did not involve wild animals.
Reporting on sex	No sex was considered in the study design, because we were just using HFD mouse serum, and it had nothing to do with sex.
Field-collected samples	This study did not involve samples collected from the field.
Ethics oversight	All animal experiments and procedures were approved by the Institutional Animal Care and Use Committee (IACUC) of the Institute of Zoology (Chinese Academy of Sciences, IOZ-IACUC-2022-170).

Note that full information on the approval of the study protocol must also be provided in the manuscript.

Plants

Seed stocks	*Report on the source of all seed stocks or other plant material used. If applicable, state the seed stock centre and catalogue number. If plant specimens were collected from the field, describe the collection location, date and sampling procedures.*
Novel plant genotypes	*Describe the methods by which all novel plant genotypes were produced. This includes those generated by transgenic approaches, gene editing, chemical/radiation-based mutagenesis and hybridization. For transgenic lines, describe the transformation method, the number of independent lines analyzed and the generation upon which experiments were performed. For gene-edited lines, describe the editor used, the endogenous sequence targeted for editing, the targeting guide RNA sequence (if applicable) and how the editor was applied.*
Authentication	*Describe any authentication procedures for each seed stock used or novel genotype generated. Describe any experiments used to assess the effect of a mutation and, where applicable, how potential secondary effects (e.g. second site T-DNA insertions, mosiacism, off-target gene editing) were examined.*
